# Supplementary figures and images for: Gua Sha, a press-stroke treatment of the skin, boosts the immune response to intradermal vaccination
Source: PeerJ. 2016 Sep 14;4:e2451. doi: 10.7717/peerj.2451 (PMC5028785; doi:10.7717/peerj.2451)

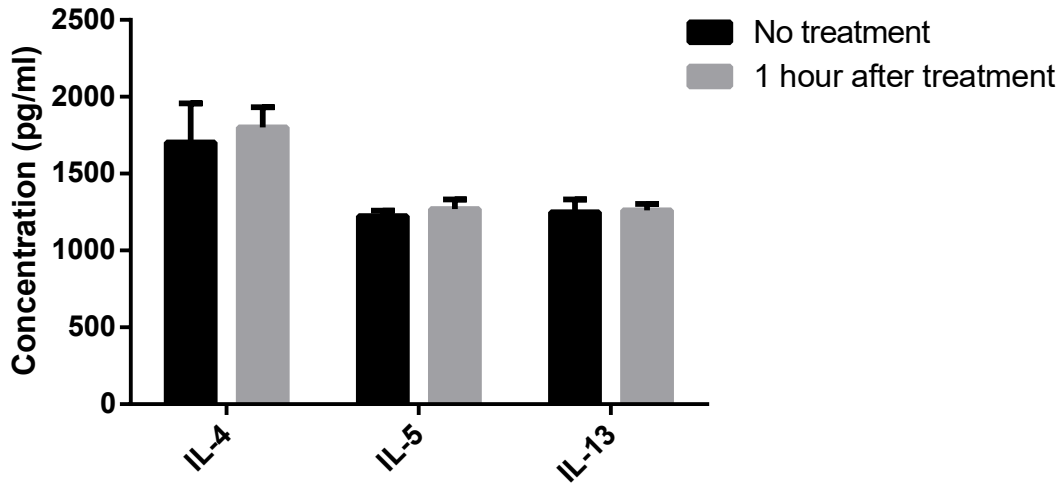

Supplement: Fig. S1 — Mice were sacrificed untreated or one hour after treatment and 0.1g of skin tissue were excised, rinsed in 1 ml PBS and homogenized on ice. The concentrations of IL-4, IL-5 and IL-13 in the supernatants were measured by ELISA and data were shown as mean + SD. Statistical comparisons were made between the treated and untreated groups. (n=6; One-way ANOVA with Dunnett’s posttest.) [file peerj-04-2451-s001.pdf]
