# Supplementary material for: Gua Sha, a press-stroke treatment of the skin, boosts the immune response to intradermal vaccination
Source: PeerJ. 2016 Sep 14;4:e2451. doi: 10.7717/peerj.2451 (PMC5028785; doi:10.7717/peerj.2451)
Supplement: Data S1 [file peerj-04-2451-s002.docx]

|  | untreated | | | | | | |
| --- | --- | --- | --- | --- | --- | --- | --- |
| IL-4 | 1652.21 | 1475.58 | 1432.75 | 1780.02 | 2134.43 | 1745.31 |  |
| IL-5 | 1278.45 | 1241.19 | 1239.54 | 1207.08 | 1173.05 | 1203.33 |  |
| IL-13 | 1264.12 | 1337.89 | 1199.76 | 1331.28 | 1119.54 | 1249.36 |  |
| (pg/ml) | 1 hour after treatment | | | | | | |
| IL-4 | 1594.88 | 1785 | 1791.04 | 1912.4 | 1971.43 | 1752.73 |  |
| IL-5 | 1241.21 | 1349.08 | 1285.3 | 1322.89 | 1246.55 | 1180.2 |  |
| IL-13 | 1290.94 | 1190.09 | 1270.77 | 1293.32 | 1295 | 1232.66 |  |
