# Supplementary material for: Gua Sha, a press-stroke treatment of the skin, boosts the immune response to intradermal vaccination
Source: PeerJ. 2016 Sep 14;4:e2451. doi: 10.7717/peerj.2451 (PMC5028785; doi:10.7717/peerj.2451)
Supplement: Data S2 [file peerj-04-2451-s003.docx]

| CD11c |  |  |  |  |
| --- | --- | --- | --- | --- |
|  | untreated | 15min | 30min | 60min |
|  | 4.18 | 8.59 | 13.7 | 14.2 |
|  | 4.69 | 8.76 | 12.2 | 13.1 |
|  | 4.93 | 9.4 | 10.6 | 14.5 |
| F4/80 |  |  |  |  |
|  | untreated | 15min | 30min | 60min |
|  | 16.5 | 17.5 | 18.4 | 19.5 |
|  | 15 | 17.8 | 18.8 | 19.4 |
|  | 15.7 | 17.1 | 18.6 | 18.5 |
