# Supplementary material for: Gua Sha, a press-stroke treatment of the skin, boosts the immune response to intradermal vaccination
Source: PeerJ. 2016 Sep 14;4:e2451. doi: 10.7717/peerj.2451 (PMC5028785; doi:10.7717/peerj.2451)
Supplement: Data S3 [file peerj-04-2451-s004.docx]

| TNF-α | untreated | scrape 1h | no scrape 1h | scrape 2h | no scrape 2h |
| --- | --- | --- | --- | --- | --- |
| (ng/ml) | 391.64 | 773.45 | 613.45 | 1064.36 | 1253.45 |
|  | 402.55 | 748.00 | 664.36 | 929.82 | 1446.18 |
|  | 369.82 | 940.73 | 908.00 | 1038.91 | 918.91 |
|  | 329.82 | 864.36 | 478.91 | 973.45 | 595.27 |
|  | 286.18 | 795.27 | 617.09 | 1075.27 | 1442.55 |
|  | 537.09 | 857.09 | 562.55 | 1006.18 | 1009.82 |
|  |  |  |  |  |  |
| IL-1β | untreated | scrape 1h | no scrape 1h | scrape 2h | no scrape 2h |
| (ng/ml) | 40.89 | 29.17 | 38.03 | 45.46 | 47.46 |
|  | 45.17 | 22.31 | 46.89 | 37.17 | 56.60 |
|  | 31.46 | 31.46 | 32.31 | 42.31 | 41.17 |
|  | 26.03 | 43.46 | 38.60 | 38.89 | 30.03 |
|  | 24.31 | 39.74 | 56.89 | 33.17 | 56.89 |
|  | 22.89 | 42.60 | 41.17 | 41.74 | 43.46 |
|  |  |  |  |  |  |
| IL-6 | untreated | scrape 1h | no scrape 1h | scrape 2h | no scrape 2h |
| (pg/ml) | 353.06 | 580.56 | 264.31 | 909.31 | 563.06 |
|  | 203.69 | 433.06 | 162.44 | 485.56 | 363.06 |
|  | 324.31 | 723.06 | 381.19 | 602.44 | 368.69 |
|  | 429.31 | 698.69 | 340.56 | 536.81 | 288.69 |
|  | 278.06 | 631.81 | 350.56 | 891.19 | 374.94 |
|  | 228.69 | 581.19 | 284.31 | 737.44 | 413.06 |
|  |  |  |  |  |  |
| IL-10 | untreated | scrape 1h | no scrape 1h | scrape 2h | no scrape 2h |
| (pg/ml) | 2238.37 | 1107.76 | 1817.96 | 1240.41 | 1748.57 |
|  | 1930.20 | 1283.27 | 1589.39 | 1189.39 | 1709.80 |
|  | 2264.90 | 1220.00 | 1866.94 | 1093.47 | 2044.49 |
|  | 2226.12 | 1177.14 | 1464.90 | 1150.61 | 1209.80 |
|  | 2062.86 | 1103.67 | 2144.49 | 1136.33 | 1583.27 |
|  | 1973.06 | 1103.67 | 1748.57 | 1938.37 | 1750.61 |
|  |  |  |  |  |  |
| IL-12p70 | untreated | scrape 1h | no scrape 1h | scrape 2h | no scrape 2h |
| (pg/ml) | 636.43 | 765.00 | 474.52 | 805.48 | 519.76 |
|  | 572.14 | 886.43 | 710.24 | 974.52 | 460.24 |
|  | 665.00 | 712.62 | 703.10 | 910.24 | 765.00 |
|  | 519.76 | 693.57 | 572.14 | 829.29 | 686.43 |
|  | 462.62 | 898.33 | 676.91 | 617.38 | 460.24 |
|  | 493.57 | 705.48 | 412.62 | 1020.71 | 717.38 |
|  |  |  |  |  |  |
| IL-23 | untreated | scrape 1h | no scrape 1h | scrape 2h | no scrape 2h |
| (pg/ml) | 444.71 | 543.24 | 459.41 | 572.65 | 409.41 |
|  | 309.41 | 431.47 | 415.29 | 452.06 | 381.47 |
|  | 444.71 | 569.71 | 355.00 | 491.76 | 488.82 |
|  | 371.18 | 466.76 | 452.06 | 525.59 | 444.41 |
|  | 381.47 | 499.12 | 343.24 | 580.00 | 343.24 |
|  | 332.94 | 502.06 | 368.24 | 547.65 | 541.76 |
