# Supplementary material for: Gua Sha, a press-stroke treatment of the skin, boosts the immune response to intradermal vaccination
Source: PeerJ. 2016 Sep 14;4:e2451. doi: 10.7717/peerj.2451 (PMC5028785; doi:10.7717/peerj.2451)
Supplement: Data S4 [file peerj-04-2451-s005.docx]

|  |  |  |  |  |  |
| --- | --- | --- | --- | --- | --- |
| TNF-α | 0.5h |  | 1h |  |  |
| pg/ml | untreated | 40 scrapes | untreated | 40 scrapes |  |
|  | 0.782644 | 70.69379 | 7.456333 | 413.5794 |  |
|  | 0 | 30.78368 | 5.645624 | 283.6894 |  |
|  | 5.987651 | 11.6833 | 9.827253 | 206.6667 |  |
|  | 0 | 35.5783 | 5.887575 | 558.3333 |  |
|  | 1.993079 | 56.56937 | 9.002682 | 516.6667 |  |
|  | 0 | 8.579375 | 6.2902 | 291.6667 |  |
|  |  |  |  |  |  |
| IL-1β | 0.5h |  | 1h |  |  |
| pg/ml | untreated | 40 scrapes | untreated | 40 scrapes |  |
|  | 0 | 0 | 5.792745 | 5.789785 |  |
|  | 0 | 7.937897 | 1.479265 | 41.57895 |  |
|  | 0 | 2.783975 | 0 | 26.65366 |  |
|  | 0 | 0 | 6.56826 | 30.48788 |  |
|  | 0 | 0 | 0 | 46.66667 |  |
|  | 0 | 27.59376 | 0 | 73.3333 |  |
|  |  |  |  |  |  |
| IL-6 | 0.5h |  | 1h |  |  |
| pg/ml | untreated | 40 scrapes | untreated | 40 scrapes |  |
|  | 1.794789 | 2.57839 | 2.56835 | 13.15355 |  |
|  | 0 | 0 | 1.793725 | 9.328765 |  |
|  | 0.3478965 | 4.639755 | 4.75892 | 12.91567 |  |
|  | 1.089275 | 0 | 2.5782 | 11.37875 |  |
|  | 1.917672 | 0.478375 | 0.46825 | 6.346784 |  |
|  | 0 | 0.872896 | 1.084625 | 15.58639 |  |
|  |  |  |  |  |  |
| NO | 0.5h |  | 1h |  |  |
| nmol/L | untreated | 40 scrapes | untreated | 40 scrapes |  |
|  | 30.38683 | 50.68685 | 47.7927 | 72.64477 |  |
|  | 43.57984 | 38.57984 | 40.74693 | 68.65875 |  |
|  | 49.58984 | 48.43866 | 43.79427 | 58.5685 |  |
|  | 48.38683 | 40.79567 | 45.89274 | 66.75879 |  |
|  | 30.78899 | 42.57894 | 38.17941 | 74.64474 |  |
|  | 34.34454 | 55.36837 | 42.34709 | 78.37894 |  |
