# Supplementary material for: Gua Sha, a press-stroke treatment of the skin, boosts the immune response to intradermal vaccination
Source: PeerJ. 2016 Sep 14;4:e2451. doi: 10.7717/peerj.2451 (PMC5028785; doi:10.7717/peerj.2451)
Supplement: Data S5 [file peerj-04-2451-s006.docx]

|  |  |  |  |  |
| --- | --- | --- | --- | --- |
| Prime IgG | OVA | OVA+20 scrapes | OVA+40 scrapes | OVA+FIA |
|  | 1.94 | 2.31 | 2.05 | 2.89 |
|  | 2.11 | 2.25 | 2.32 | 2.82 |
|  | 2.51 | 2.19 | 2.45 | 2.42 |
|  | 2.03 | 2.29 | 2.47 | 2.21 |
|  | 2.35 | 2.24 | 2.17 | 2.75 |
|  | 2.42 | 2.12 | 2.56 | 2.83 |
|  |  |  |  |  |
| 1st boost IgG | OVA | OVA+20 scrapes | OVA+40 scrapes | OVA+FIA |
|  | 3.75 | 4.02 | 4.06 | 5.18 |
|  | 3.42 | 3.88 | 4.29 | 4.92 |
|  | 3.75 | 3.92 | 4.05 | 5.22 |
|  | 3.37 | 4.02 | 4.17 | 5.11 |
|  | 3.85 | 4.23 | 4.57 | 5.21 |
|  | 3.87 | 3.87 | 3.74 | 4.99 |
|  |  |  |  |  |
| 2nd boost IgG | OVA | OVA+20 scrapes | OVA+40 scrapes | OVA+FIA |
|  | 4.40 | 4.87 | 4.90 | 5.14 |
|  | 4.74 | 4.95 | 4.99 | 5.03 |
|  | 4.49 | 4.73 | 5.27 | 5.24 |
|  | 4.60 | 4.84 | 4.99 | 5.20 |
|  | 4.31 | 4.74 | 5.02 | 5.30 |
|  | 4.59 | 5.05 | 4.87 | 5.12 |
|  |  |  |  |  |
| 2nd boost IgG1 | OVA | OVA+20 scrapes | OVA+40 scrapes | OVA+FIA |
|  | 4.47 | 4.81 | 4.67 | 5.10 |
|  | 4.41 | 4.51 | 4.87 | 4.89 |
|  | 4.48 | 4.46 | 4.99 | 4.91 |
|  | 4.53 | 4.74 | 4.49 | 4.74 |
|  | 4.18 | 4.81 | 4.57 | 4.91 |
|  | 4.40 | 5.03 | 4.64 | 4.99 |
|  |  |  |  |  |
| 2nd boost IgG2a | OVA | OVA+20 scrapes | OVA+40 scrapes | OVA+FIA |
|  | 1.00 | 3.25 | 3.78 | 2.96 |
|  | 3.09 | 3.28 | 3.59 | 3.24 |
|  | 1.00 | 3.31 | 3.64 | 3.32 |
|  | 2.93 | 3.41 | 3.45 | 3.25 |
|  | 1.00 | 3.47 | 3.30 | 3.37 |
|  | 3.07 | 3.47 | 3.57 | 3.51 |
|  |  |  |  |  |
| 2nd boost IgG1/IgG2a | OVA | OVA+20 scrapes | OVA+40 scrapes | OVA+FIA |
|  |  | 1.48 | 1.24 | 1.72 |
|  | 1.43 | 1.38 | 1.36 | 1.51 |
|  |  | 1.35 | 1.37 | 1.48 |
|  | 1.55 | 1.39 | 1.30 | 1.46 |
|  |  | 1.39 | 1.39 | 1.46 |
|  | 1.43 | 1.45 | 1.30 | 1.42 |
